# Mediation of the frailty index, the relationship between sleep duration and chronic pain in different body regions: insights from the CHARLS cross-sectional study

**DOI:** 10.1186/s13018-025-06157-5

**Published:** 2025-08-06

**Authors:** Changqing Li, Hong Ding, Lei Zhang, Chao Ma, Caiyun Ma, Hebao Wen, Xiaojiang Zhao

**Affiliations:** 1 Department of Physical Education and Arts, Bengbu Medical University, Bengbu, 233000 China; 2 Anhui Engineering Research Center for Neural Regeneration Technology and Medical New Materials, Bengbu Medical University, Bengbu, 233000 China

**Keywords:** Cross-sectional study, Chronic pain, Sleep duration, Frailty index

## Abstract

**Background:**

Sleep duration and frailty index are knowm to influence chronic pain. However, the manner in which these factors interact to influence chronic pain remains unclear. The present study investigates the link between sleep duration and chronic pain using a cross-sectional design while also examining the potential mediating role of the frailty index.

**Methods:**

This study incorporated 2015 China Health and Retirement Longitudinal Study (CHARLS) data in a cross-sectional design. Multivariate logistic regression was used to assess the link between sleep and chronic pain, and mediation analysis was used to explore the role played by the frailty index.

**Results:**

The study comprised 10,321 individuals who were at least 45 years old, of whom 3,051 had at least one site of chronic pain. Seep duration was negatively associated with chronic pain in different body regions. The frailty index was positively associated with these sites, and it mediated the associations between sleep duration and chronic pain in different body regions.

**Conclusion:**

Sleep duration is negatively correlated with chronic pain, whereas the frailty index is positively correlated with chronic pain in different body regions, potentially serving as a mediating factor. This study highlights the importance of incorporating both sleep duration and the frailty index into chronic pain management strategies.

**Supplementary Information:**

The online version contains supplementary material available at 10.1186/s13018-025-06157-5.

## Introduction

Chronic pain is a complex disorder that can be caused by a variety of factors, and it is characterized by persistent or recurring chronic pain that lasts longer than 3 months [1, 2]. Chronic pain is becoming more common in China annually, and it has an effect on an expanding group of middle-aged and older adults [3]. Long-term chronic pain can cause depression, disability, and a lower quality of life, resulting in significant distress to patients [4]. By 2025, 20% of China’s population will be 60 years or older, significantly straining both the health care system and society [5]. Additionally, chronic pain exerts a substantial economic burden on a global scale. According to conservative estimates, European countries alone spent $185 billion on pain management in 2014 [6]. The burden of chronic pain for both health care and society at large is increasing worldwide. While the precise mechanisms underlying chronic pain remain unclear, the identified risk factors include getting poor sleep, being overweight, and engaging in certain lifestyle choices [7].

Sleep is vital for health, and studies have linked sleep duration to chronic pain [8]. One study showed that insomnia, or sleep deprivation, increased the risk of chronic pain, and poor sleep significantly predicted more severe chronic pain [9]. In addition, there is neurobiological evidence that sleep deprivation plays a role in the regulation of chronic pain. Biological, psychological, and social possibilities should be noted in the relationship between sleep and chronic pain [10]. Another study revealed a bidirectional relationship between sleep and chronic pain [11]. Sleep problems can exacerbate the degree of chronic pain, and chronic pain can, in turn, lead to a decline in sleep quality [12, 13]. This interaction may work through multiple mechanisms, including neurobiochemical pathways, inflammatory responses, and psychological factors [14]. There is neurobiological evidence showing that sleep deprivation plays a role in the regulation of chronic pain. The biological, psychological, and social possibilities concerning the relationship between sleep and chronic pain should be noted [10].

Frailty, which is a growing geriatric syndrome characterized by the deterioration of various physiological systems, presents a major challenge for global health care [15, 16]. The frailty index is frequently used to evaluate frailty status. A higher frailty index value is linked to adverse health outcomes, including disability, limited mobility, disease, hospitalization, and increased mortality [17–19]. It also represents a risk factor for chronic pain [20]. Several previous studies have shown that sleep duration and frailty exert significant effects on pain [11–23]. Additionally, sleep duration is correlated with the frailty index. One study suggested that people aged over 45 years face increasing health challenges as they age and their physical function declines, with sleep disorders and frailty being common problems [24]. A recent cross-sectional study set among a Chinese population revealed a correlation among sleep duration, the frailty index and chronic pain [25]. However, establishing a causal link among sleep duration, the frailty index, and chronic pain is complex because of the interaction of factors and potential reverse causation.

We preliminarily analysed the relationship between sleep duration and chronic pain in different body regions using a cross-sectional study that used data from the 2015 China Health and Retirement Longitudinal Study (CHARLS) and explored whether the frailty index plays a mediating role. These findings may have important implications for clinical practice.

## Methods

### Study design and population

**Fig. 1:**
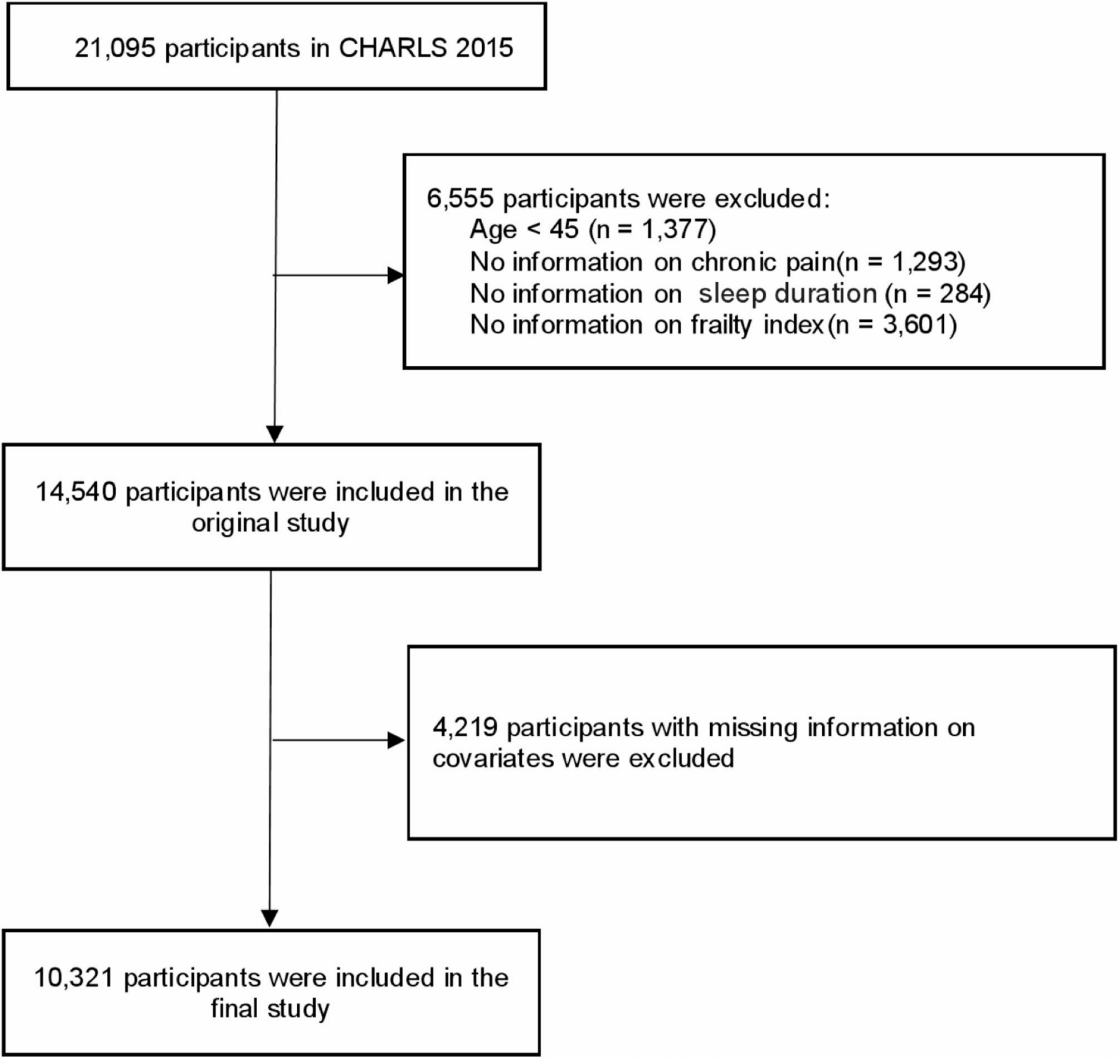
Flowchart of the participant selection process Abbreviations: CHARLS, China Health and Retirement Longitudinal Study

The CHARLS served as the data source for this cross-sectional study. The CHARLS is a longitudinal, sample of robust size, and it is a nationally representative survey of Chinese adults aged 45 years and above that was designed to create a comprehensive, publicly accessible microdata set that is both reliable and representative. A multistage, stratified sampling approach was used, in which 450 primary units from 150 counties or districts were selected on the basis of size. The baseline survey spanned the period of 2011–2012, with follow-ups in 2013, 2015, 2018, and 2020. The detailed survey methods used are available in previous studies [26]. The Ethics Committee of Peking University (IRB00001052-11015) approved the CHARLS, and all participants provided written consent. In this study, we used data from the third wave (2015) of the follow-up survey. The initial cohort consisted of 21,095 respondents, and to ensure analytic rigor, exclusion criteria were applied sequentially, as follows: individuals under 45 years of age (*n* = 1,377), those with unrecorded pain (*n* = 1,293) (lack of any pain-related data), the lack of sleep duration (*n* = 284), an incomplete frailty index assessment in individuals (*n* = 3,601) and the lack of covariate information (*n* = 4,219). Among the 21,095 initially enrolled participants, 10,774 were excluded. This represented 51.07% of the original cohort, leaving 10,321 participants (48.93%) for the final analysis. Figure 1 illustrates the study population selection process.

### Assessment of sleep duration, frailty index and chronic pain in different body regions

In this study, chronic pain served as the dependent variable, whereas sleep duration and the frailty index were used as the independent variables. Sleep duration data was collected from the CHARLS questionnaire’s lifestyle and health behaviour section, in which participants reported their average nightly sleep over the past month in hours and minutes. In accordance with a previous study and the American Academy of Sleep Medicine’s guidelines [27], the participants were categorized into three sleep duration groups: short (< 6 h/day), normal (6–8 h/day), and long ( ≧ 8 h/day). Participants whose average sleep duration was < 3 h or > 11 h were excluded to minimize extreme outliers [28]. The frailty of the participants was assessed using their frailty index scores, which is more effective than using chronological age for capturing age-related health declines [29]. This index provides a comprehensive evaluation of older adults’ health, particularly for assessing adverse outcomes. Despite variations in the indicators used across studies, key health domains such as daily activities, chronic diseases, psychological traits, and cognitive function were consistently included. This study used a standardized procedure to calculate the frailty index, selecting 32 items including comorbidities, physical function, disabilities, depression, and cognition [30]. The frailty index, which was validated using the CHARLS database and other cohorts, consists of 32 items [31–33]. Each item, except for the 32nd item, is scored as 0 (no deficit) or 1 (deficit present). The 32nd item is a continuous variable ranging from 0 to 1, and it indicates cognitive decline. The details are provided in Supplemental Table 1. The index is calculated by summing the health deficits and dividing by 32, which results in a score between 0 and 1, with higher scores indicating greater frailty. To handle any missing data, up to 20% of the missing variables (≤ 6 items) were allowed [30]. If more than 6 items were missing, the index was considered missing. Otherwise, the average of nonmissing values was used.

In this study, chronic pain referred to chronic pain in different body regions. Data concerning chronic pain in different body regions were obtained from the lifestyle and health behaviours section of the CHARLS questionnaire. CHARLS investigators used a self-report questionnaire to collect information on body pain during the data collection process. The first question of the questionnaire was “Are you often bothered by physical pain?” Pain was classified as a binary variable (yes/no) according to participant responses, with pain areas identified by pain sites. Chronic pain in different body regions included headaches, shoulder pain, arm pain, wrist pain, finger pain, chest pain, stomach aches, back pain, waist pain, buttock pain, leg pain, knee pain, ankle pain, toe pain, and neck pain.

### Covariates

This study incorporated sociodemographic characteristics, lifestyle habits, and personal health status as covariates. The sociodemographic variables included age, sex, educational attainment, marital status, and usual residence. Sex was categorized as male or female. Educational attainment was classified into three levels: “illiteracy,” “primary or middle school,” and “high school and above.” Marital status was defined as “married” for individuals cohabitating with a spouse and “unmarried” for those who were separated, divorced, widowed, or single. Usual residence was dichotomized into rural or urban categories. Lifestyle habits included smoking and drinking status, with each classified as “yes” or “no.” Personal health status was assessed using body mass index (BMI) and 14 self-reported noncommunicable conditions, which included hypertension, dyslipidaemia, diabetes, cancer, chronic pulmonary disease, liver disease, myocardial infarction, cerebrovascular accidents, renal disease, asthma, psychiatric disorders, gastrointestinal disease, cognitive disorders, and arthritis.

### Statistical methods

In the cross-sectional study, all analyses were conducted using R 4.3.3 and Free Statistics software 2.0. The sample characteristics are described using the tableone package (version 0.13.2). The continuous variables are summarized using means and standard deviations, whereas categorical variables are summarized using frequencies and percentages. Spearman’s correlation was used to assess the relationships between the main variables. Chronic pain in different body regions was classified as a binary variable (yes/no), so logistic regression was used. The odds ratio (OR) was used to quantify the magnitude of the associations between variables. Logistic regression analysis was conducted using the R software package glmnet (version 4.1–8). Multivariate logistic regression was used for covariate adjustment. Four asymptotic adjustment models were used, as follows: an unadjusted model, Model 1 (age, sex, educational level, and marital status), Model 2 (Model 1 + smoking status, drinking status, and BMI), and Model 3 (adjusted for all covariates). Second, using the adjustment variables in Model 3, we applied restricted cubic spline (RCS) fitting to investigate any potential nonlinear associations between sleep duration and chronic pain in different body regions. RCS analyses were performed by the rms package (version 5.1–4) in R software, and the locations of the knots were set at 2, 4, 6, 8, 10, and 12 for a total of six knots. When a nonlinear correlation was identified, a two-piecewise regression model was employed to ascertain the presence of a threshold effect. Following the detection of a threshold effect, we determined the inflection point and computed the OR with 95% CI for the segments and right of the inflection point (IP). Likelihood ratio tests (LRTs) were conducted to assess the threshold effects of sleep duration and chronic pain in different body regions. Finally, we performed moderated mediation analyses using the mediation package (version 4.5.0) in R to test for mediation [34]. The frailty index plays a mediating role in the pathway of the causal association between sleep duration and chronic pain in different body regions. This mediator effect was measured by determining the mediator percentage, and its significance was assessed through bootstrap resampling (times = 1000). P values < 0.05 were considered statistically significant. The Bonferroni correction was applied to minimize Type I errors, which were calculated as 0.05 divided by the number of subsamples [35]. In this instance, with chronic pain present in 15 body regions, the correction was 0.05/15. Therefore, only variables with p values less than 0.0033 were considered.

## Results

### Baseline characteristics of the study population

Table 1 presents the characteristics of the participants as categorized by sleep duration. Among the 10,321 participants, 3,070 reported sleeping for less than 6 h, 4,158 reported sleeping 6–8 h, and 3,093 reported sleeping for more than 8 h. Chronic pain in different body regions differed significantly across sleep duration subgroups (*p* < 0.001). The participants’ average age was 59.3 ± 9.3 years, and 5,254 (50.9%) were female. Compared with those in the other groups, those with a sleep duration of 6–8 h were more likely to be younger on average; have a lower frailty index; be male; be nonsmokers; and be nondrinkers. In addition, the three groups differed significantly (*p* < 0.001) in terms of residence, marital status, education level, the presence of 14 chronic diseases, and having chronic pain in different body regions.

**Table 1:** Characteristics of the participants by the sleep duration groups

Variables	Total	Sleep duration per night in hours			*P*
		< 6 h	6–8 h	> 8 h	
	10,321	3070	4158	3093	
Age, Mean ± SD	59.3 ± 9.3	60.8 ± 9.4	58.3 ± 8.8	59.4 ± 9.5	< 0.001
Sex, n (%)					< 0.001
Female	5254 (50.9)	1803 (58.7)	1920 (46.2)	1531 (49.5)	
Male	5067 (49.1)	1267 (41.3)	2238 (53.8)	1562 (50.5)	
Residence, n (%)					< 0.001
Rural	6379 (61.8)	1970 (64.2)	2385 (57.4)	2024 (65.4)	
Urban	3942 (38.2)	1100 (35.8)	1773 (42.6)	1069 (34.6)	
Marital_status, n (%)					< 0.001
Married and living with a spouse	8804 (85.3)	2503 (81.5)	3636 (87.4)	2665 (86.2)	
Married but living without a spouse	454 ( 4.4)	142 (4.6)	183 (4.4)	129 (4.2)	
Single, divorced, and windowed	1063 (10.3)	425 (13.8)	339 (8.2)	299 (9.7)	
Education_Status, n (%)					< 0.001
Elementary school or below	7012 (67.9)	2246 (73.2)	2583 (62.1)	2183 (70.6)	
Middle school or above	3309 (32.1)	824 (26.8)	1575 (37.9)	910 (29.4)	
Smoking_Status, n (%)					< 0.001
Non-smoker	5733 (55.5)	1825 (59.4)	2190 (52.7)	1718 (55.5)	
Smoker	4588 (44.5)	1245 (40.6)	1968 (47.3)	1375 (44.5)	
Drinking_Status, n (%)					< 0.001
Drink but less than once a month	912 ( 8.8)	255 (8.3)	405 (9.7)	252 (8.1)	
Drink more than once a month	2897 (28.1)	761 (24.8)	1274 (30.6)	862 (27.9)	
Non-drinker	6512 (63.1)	2054 (66.9)	2479 (59.6)	1979 (64)	
BMI, Mean ± SD	24.7 ± 17.5	24.8 ± 19.0	24.7 ± 16.0	24.7 ± 18.0	0.958
14 chronic diseases, n (%)					< 0.001
0	3045 (29.5)	705 (23)	1299 (31.2)	1041 (33.7)	
1	2397 (23.2)	633 (20.6)	999 (24)	765 (24.7)	
≧ 2	4879 (47.3)	1732 (56.4)	1860 (44.7)	1287 (41.6)	
Headache pain, n (%)					< 0.001
No	8830 (85.6)	2345 (76.4)	3725 (89.6)	2760 (89.2)	
Yes	1491 (14.4)	725 (23.6)	433 (10.4)	333 (10.8)	
Shoulder pain, n (%)					< 0.001
No	8820 (85.5)	2357 (76.8)	3691 (88.8)	2772 (89.6)	
Yes	1501 (14.5)	713 (23.2)	467 (11.2)	321 (10.4)	
Arm pain, n (%)					< 0.001
No	9103 (88.2)	2482 (80.8)	3775 (90.8)	2846 (92)	
Yes	1218 (11.8)	588 (19.2)	383 (9.2)	247 (8)	
Wrist pain, n (%)					< 0.001
No	9435 (91.4)	2631 (85.7)	3900 (93.8)	2904 (93.9)	
Yes	886 ( 8.6)	439 (14.3)	258 (6.2)	189 (6.1)	
Fingers pain, n (%)					< 0.001
No	9400 (91.1)	2602 (84.8)	3890 (93.6)	2908 (94)	
Yes	921 ( 8.9)	468 (15.2)	268 (6.4)	185 (6)	
Chest pain, n (%)					< 0.001
No	9628 (93.3)	2708 (88.2)	3965 (95.4)	2955 (95.5)	
Yes	693 ( 6.7)	362 (11.8)	193 (4.6)	138 (4.5)	
Stomachache, n (%)					< 0.001
No	9294 (90.0)	2581 (84.1)	3835 (92.2)	2878 (93)	
Yes	1027 (10.0)	489 (15.9)	323 (7.8)	215 (7)	
Back pain, n (%)					< 0.001
No	9160 (88.8)	2524 (82.2)	3804 (91.5)	2832 (91.6)	
Yes	1161 (11.2)	546 (17.8)	354 (8.5)	261 (8.4)	
Waist pain, n (%)					< 0.001
No	8270 (80.1)	2141 (69.7)	3505 (84.3)	2624 (84.8)	
Yes	2051 (19.9)	929 (30.3)	653 (15.7)	469 (15.2)	
Buttocks pain, n (%)					< 0.001
No	9718 (94.2)	2772 (90.3)	3985 (95.8)	2961 (95.7)	
Yes	603 ( 5.8)	298 (9.7)	173 (4.2)	132 (4.3)	
Leg pain, n (%)					< 0.001
No	8738 (84.7)	2332 (76)	3672 (88.3)	2734 (88.4)	
Yes	1583 (15.3)	738 (24)	486 (11.7)	359 (11.6)	
Knees pain, n (%)					< 0.001
No	8675 (84.1)	2302 (75)	3641 (87.6)	2732 (88.3)	
Yes	1646 (15.9)	768 (25)	517 (12.4)	361 (11.7)	
Ankle pain, n (%)					< 0.001
No	9496 (92.0)	2654 (86.4)	3922 (94.3)	2920 (94.4)	
Yes	825 ( 8.0)	416 (13.6)	236 (5.7)	173 (5.6)	
Toes pain, n (%)					< 0.001
No	9798 (94.9)	2800 (91.2)	4011 (96.5)	2987 (96.6)	
Yes	523 ( 5.1)	270 (8.8)	147 (3.5)	106 (3.4)	
Neck pain, n (%)					< 0.001
No	9312 (90.2)	2596 (84.6)	3834 (92.2)	2882 (93.2)	
Yes	1009 ( 9.8)	474 (15.4)	324 (7.8)	211 (6.8)	
Frailty index, Mean ± SD	0.2 ± 0.1	0.2 ± 0.1	0.1 ± 0.1	0.1 ± 0.1	< 0.001

Abbreviations: BMI, body mass index

### Associations among sleep duration, frailty index and chronic pain in different body regions

Table 2 shows the correlations between sleep duration and chronic pain in different body regions. An analysis of the unadjusted model revealed statistically significant negative correlations between sleep duration and chronic pain in different body regions (all *p* < 0.001), which remained significant after Bonferroni correction for 15 tests. The correlations also remained consistent when the covariates were considered in Models 1, 2, and 3. The relationships between the frailty index and chronic pain in different body regions remained significantly positive in both the unadjusted model and the three progressively adjusted models (Table 3) (all *p* < 0.001), which remained significant after Bonferroni correction for 15 tests. The correlations among sleep duration, the frailty index, and chronic pain in different body regions are displayed in Supplemental Table 1. We found that sleep duration was negatively correlated with the frailty index. Sleep duration was negatively correlated with chronic pain in different body regions, and the frailty index was positively correlated with chronic pain in different body regions.

**Table 2:** Associations of sleep duration with chronic pain in different body regions

Location	No	Unadjusted	Model 1	Model 2	Model 3
		OR (95% CI)	OR (95% CI)	OR (95% CI)	OR (95% CI)
Headache	2176	0.78 (0.75 ~ 0.80)***	0.84 (0.81 ~ 0.86)***	0.84 (0.81 ~ 0.86)***	0.84 (0.81 ~ 0.86)***
Shoulder pain	2166	0.79 (0.76 ~ 0.81)***	0.84 (0.82 ~ 0.87)***	0.84 (0.82 ~ 0.87)***	0.84 (0.82 ~ 0.87)***
Arm pain	1733	0.78 (0.75 ~ 0.80)***	0.84 (0.81 ~ 0.87)***	0.84 (0.81 ~ 0.87)***	0.84 (0.81 ~ 0.87)***
Wrist pain	1271	0.78 (0.75 ~ 0.81)***	0.85 (0.82 ~ 0.88)***	0.85 (0.82 ~ 0.88)***	0.85 (0.82 ~ 0.88)***
Fingers pain	1308	0.76 (0.74 ~ 0.79)***	0.84 (0.81 ~ 0.87)***	0.84 (0.81 ~ 0.87)***	0.84 (0.81 ~ 0.87)***
Chest pain	974	0.77 (0.74 ~ 0.80)***	0.84 (0.81 ~ 0.88)***	0.84 (0.81 ~ 0.88)***	0.84 (0.81 ~ 0.88)***
Stomachache	1479	0.79 (0.76 ~ 0.81)***	0.85 (0.82 ~ 0.88)***	0.85 (0.83 ~ 0.88)***	0.85 (0.83 ~ 0.88)***
Back pain	1678	0.80 (0.77 ~ 0.83)***	0.87 (0.84 ~ 0.90)***	0.87 (0.84 ~ 0.90)***	0.87 (0.84 ~ 0.90)***
Waist pain	3021	0.80 (0.78 ~ 0.82)***	0.85 (0.83 ~ 0.87)***	0.85 (0.83 ~ 0.87)***	0.85 (0.83 ~ 0.87)***
Buttocks pain	878	0.78 (0.75 ~ 0.81)***	0.85 (0.82 ~ 0.89)***	0.85 (0.83 ~ 0.89)***	0.85 (0.82 ~ 0.89)***
Leg pain	2266	0.80 (0.78 ~ 0.82)***	0.86 (0.84 ~ 0.89)***	0.86 (0.84 ~ 0.89)***	0.86 (0.84 ~ 0.89)***
Knees pain	2363	0.79 (0.77 ~ 0.82)***	0.86 (0.83 ~ 0.88)***	0.86 (0.83 ~ 0.88)***	0.86 (0.83 ~ 0.88)***
Ankle pain	1178	0.77 (0.74 ~ 0.80)***	0.85(0.82 ~ 0.88)***	0.85 (0.81 ~ 0.88)***	0.85 (0.81 ~ 0.88)***
Toes pain	772	0.76 (0.73 ~ 0.80)***	0.85 (0.81 ~ 0.89)***	0.85 (0.81 ~ 0.89)***	0.85 (0.81 ~ 0.89)***
Neck pain	1457	0.80 (0.77 ~ 0.82)***	0.86 (0.83 ~ 0.89)***	0.86 (0.83 ~ 0.89)***	0.86 (0.83 ~ 0.89)***

Model 1: adjusted for age, gender, educational level, and marital status. Model 2: adjusted for model 1 + smoking status, drinking status, and BMI. Model 3: adjusted for model 2 + 14 chronic diseases. Abbreviations: OR, odds ratio; 95% CI, 95% confidence interval. *** P-value < 0.001. Goodness-of-fit test: *p* = 0.56

**Table 3:** Associations of frailty index with chronic pain in different body regions

Location	No	Unadjusted	Model 1	Model 2	Model 3
		OR (95% CI)	OR (95% CI)	OR (95% CI)	OR (95% CI)
Headache	2176	1.26 (1.24 ~ 1.28)***	1.23 (1.21 ~ 1.25)***	1.23 (1.21 ~ 1.25)***	1.23 (1.21 ~ 1.25)***
Shoulder pain	2166	1.26 (1.24 ~ 1.28)***	1.22 (1.21 ~ 1.26)***	1.24(1.22 ~ 1.26)***	1.24 (1.21 ~ 1.26)***
Arm pain	1733	1.26 (1.24 ~ 1.28)***	1.24 (1.24 ~ 1.27)***	1.25 (1.25 ~ 1.27)***	1.25 (1.25 ~ 1.27)***
Wrist pain	1271	1.25 (1.23 ~ 1.27)***	1.24 (1.21 ~ 1.26)***	1.24 (1.22 ~ 1.26)***	1.24 (1.22 ~ 1.26)***
Fingers pain	1308	1.27 (1.25 ~ 1.28)***	1.23 (1.21 ~ 1.24)***	1.24 (1.21 ~ 1.26)***	1.24 (1.21 ~ 1.26)***
Chest pain	974	1.25 (1.23 ~ 1.27)***	1.21 (1.18 ~ 1.23)***	1.21 (1.19 ~ 1.24)***	1.21 (1.19 ~ 1.24)***
Stomachache	1479	1.23 (1.21 ~ 1.24)***	1.18 (1.16 ~ 1.21)***	1.18 (1.16 ~ 1.20)***	1.18 (1.16 ~ 1.20)***
Back pain	1678	1.25 (1.24 ~ 1.27)***	1.23 (1.20 ~ 1.25)***	1.23 (1.21 ~ 1.25)***	1.23 (1.21 ~ 1.25)***
Waist pain	3021	1.28 (1.26 ~ 1.29)***	1.27 (1.25 ~ 1.29)***	1.27 (1.25 ~ 1.29)***	1.27 (1.25 ~ 1.29)***
Buttocks pain	878	1.25 (1.23 ~ 1.27)***	1.23 (1.20 ~ 1.25)***	1.23 (1.20 ~ 1.25)***	1.23 (1.20 ~ 1.25)***
Leg pain	2266	1.30 (1.28 ~ 1.32)***	1.29 (1.26 ~ 1.31)***	1.29 (1.27 ~ 1.31)***	1.29 (1.27 ~ 1.31)***
Knees pain	2363	1.29 (1.28 ~ 1.31)***	1.27 (1.25 ~ 1.29)***	1.27 (1.25 ~ 1.29)***	1.27 (1.25 ~ 1.29)***
Ankle pain	1178	1.28 (1.26 ~ 1.30)***	1.25 (1.23 ~ 1.28)***	1.25 (1.23 ~ 1.28)***	1.25 (1.23 ~ 1.28)***
Toes pain	772	1.28(1.25 ~ 1.30)***	1.24 (1.22 ~ 1.27)***	1.25 (1.22 ~ 1.27)***	1.25 (1.22 ~ 1.27)***
Neck pain	1457	1.24 (1.22 ~ 1.26)***	1.22 (1.20 ~ 1.24)***	1.22 (1.20 ~ 1.24)***	1.22 (1.20 ~ 1.24)***

Model 1: adjusted for age, gender, educational level, and marital status. Model 2: adjusted for model 1 + smoking status, drinking status, and BMI. Model 3: adjusted for model 2 + 14 chronic diseases. Abbreviations: OR, odds ratio; 95% CI, 95% confidence interval. *** P-value < 0.001. Goodness-of-fit test: *p* = 0.43

**Fig. 2:**
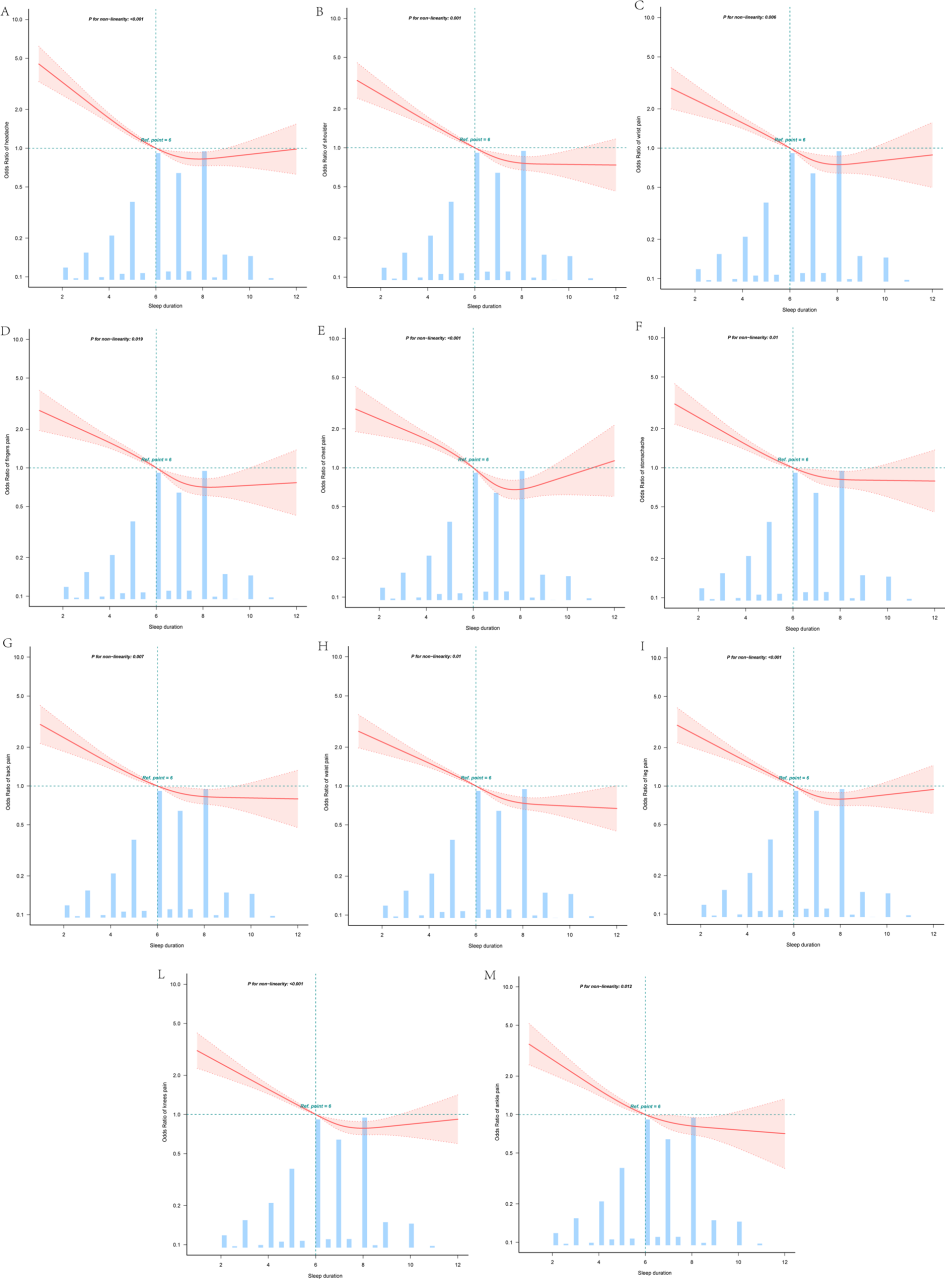
Nonlinear associations between sleep duration and chronic pain in different body regions. The solid and dashed lines represent the predicted values and 99% CIs, respectively. The orange bars represent the distribution of the entire cohort. Adjusted for age, sex, educational level, marital status, residence, smoking status, drinking status, BMI, and 14 chronic diseases, only 99% of the data are displayed.

After accounting for the confounding variables, a regression analysis with RCS revealed a nonlinear relationship between sleep duration and chronic pain in 11 areas, including the head, shoulders, wrists, fingers, chest, stomach, back, waist, legs, knees, and ankles (Fig. 2). No such relationship was found for the arms, buttocks, toes, or neck (Supplemental Fig. 1). Using a two-piecewise linear regression model, inflection points and likelihood ratio tests were calculated for the 11 chronic pain areas, all of which yielded significant results (Supplemental Tables 2–13).

The results of the bootstrap mediation test are shown in Table 4. The results reveal the mediating effect of the frailty index between sleep duration and chronic pain at 15 pain sites. Specifically, headache (mediation effect = -1.02 × 10 ^− 2^, mediated proportion = 40.48%), shoulder pain (mediation effect = -1.05 × 10^− 2^, mediated proportion = 43.57%), arm pain (mediation effect = -9.61 × 10 ^− 3^, mediated proportion = 44.69%), wrist pain (mediation effect = -7.78 × 10 ^− 3^, mediated proportion = 48.02%), finger pain (mediation effect = -8.17 × 10 ^− 3^, mediated proportion = 45.89%), chest pain (mediation effect = -6.34 × 10 ^− 3^, mediated proportion = 45.61%), stomach pain (mediation effect = -7.21 × 10 ^− 3^, mediated proportion = 41.44%), back pain (mediation effect =-9.13 × 10 ^− 3^, mediated proportion = 52.17%), waist pain (mediation effect = -1.30 × 10 ^− 2^, mediated proportion = 45.29%), buttock pain (mediation effect = -5.86 × 10 ^− 3^, mediated proportion = 50.52%), leg pain (mediation effect = -1.23 × 10 ^− 2^, mediated proportion = 52.79%), knees pain (mediation effect= -1.20 × 10 ^− 2^, mediated proportion = 48.78%), ankle pain ( mediation effect = -8.07 × 10 ^− 3^, mediated proportion = 49.81%), toes pain (mediation effect = -5.86 × 10 ^− 3^, mediated proportion = 52.32%), and neck pain ( mediation effect = 8.07 × 10 ^− 3^, mediated proportion = 48.91%).

**Table 4:** The mediation of frailty index in the relationships between sleep duration and pain in different body regions

Location	β_0_	β_1_	β_2_	β	Mediation proportion(%)
Headache	−2.52 × 10^− 2^ *P* < 0.001	-9.61 × 10^− 3^ *P* < 0.001	1.06 *P* < 0.001	-1.02 × 10^− 2^ *P* < 0.001	40.48
Shoulder pain	-2.41 × 10^− 2^ *P* < 0.001	-9.61 × 10^− 3^ *P* < 0.001	1.09 *P* < 0.001	-1.05 × 10^− 2^ *P* < 0.001	43.57
Arm pain	-2.15 × 10^− 2^ *P* < 0.001	-9.61 × 10^− 3^ *P* < 0.001	1.00 *P* < 0.001	-9.61 × 10^− 3^ *P* < 0.001	44.69
Wrist pain	-1.62 × 10^− 2^ *P* < 0.001	-9.61 × 10^− 3^ *P* < 0.001	0.81 *P* < 0.001	-7.78 × 10^− 3^ *P* < 0.001	48.02
Fingers pain	-1.78 × 10^− 2^ *P* < 0.001	-9.61 × 10^− 3^ *P* < 0.001	0.85 *P* < 0.001	-8.17 × 10^− 3^ *P* < 0.001	45.89
Chest pain	-1.39 × 10^− 2^ *P* < 0.001	-9.61 × 10^− 3^ *P* < 0.001	0.66 *P* < 0.001	-6.34 × 10^− 3^ *P* < 0.001	45.61
Stomachache	-1.74 × 10^− 2^ *P* < 0.001	-9.61 × 10^− 3^ *P* < 0.001	0.75 *P* < 0.001	-7.21 × 10^− 3^ *P* < 0.001	41.44
Back pain	-1.75 × 10^− 2^ *P* < 0.001	-9.61 × 10^− 3^ *P* < 0.001	0.95 *P* < 0.001	-9.13 × 10^− 3^ *P* < 0.001	52.17
Waist pain	-2.87 × 10^− 2^ *P* < 0.001	-9.61 × 10^− 3^ *P* < 0.001	1.35 *P* < 0.001	-1.30 × 10^− 2^ *P* < 0.001	45.29
Buttocks pain	1.16 × 10^− 2^ *P* < 0.001	-9.61 × 10^− 3^ *P* < 0.001	0.61 *P* < 0.001	-5.86 × 10^− 3^ *P* < 0.001	50.52
Leg pain	-2.33 × 10^− 2^ *P* < 0.001	-9.61 × 10^− 3^ *P* < 0.001	1.28 *P* < 0.001	-1.23 × 10^− 2^ *P* < 0.001	52.79
Knees pain	-2.46 × 10^− 2^ *P* < 0.001	-9.61 × 10^− 3^ *P* < 0.001	1.25 *P* < 0.001	-1.20 × 10^− 2^ *P* < 0.001	48.78
Ankle pain	-1.62 × 10^− 2^ *P* < 0.001	-9.61 × 10^− 3^ *P* < 0.001	0.84 *P* < 0.001	-8.07 × 10^− 3^ *P* < 0.001	49.81
Toes pain	-1.12 × 10^− 2^ *P* < 0.001	-9.61 × 10^− 3^ *P* < 0.001	0.61 *P* < 0.001	-5.86 × 10^− 3^*P* < 0.001	52.32
Neck pain	-1.65 × 10^− 2^ *P* < 0.001	-9.61 × 10^− 3^ *P* < 0.001	0.84 *P* < 0.001	8.07 × 10^− 3^ *P* < 0.001	48.91

Adjusted for age, gender, educational level, marital status, residence, annual household expenditure, smoking status, drinking status, BMI, and 14 chronic diseases. β_0_ was the total effect of sleep duration on chronic pain sites; β_1_ represents the effect of sleep duration on frailty index; β_2_ represents the effect of frailty index on chronic pain sites. β was mediation effect. The mediation effect was computed as the product of “β_1_” and “β_2_“(β_1_ × β_2_), and the mediation proportion was calculated as the ratio of the β product to total effects (β/β_0_)

## Discussion

This study employed a cross-sectional design to elucidate the relationship between sleep duration and chronic pain in different body regions and to investigate whether the frailty index plays a mediating role in this relationship. In the cross-sectional analysis, sleep duration was negatively associated with chronic pain in different body regions, whereas the frailty index was positively associated with these same sites, indicating that the frailty index mediates the association between sleep duration and chronic pain in different body regions.

In our study, sleep duration was found to be a protective factor for 15 chronic pain sites in middle-aged and older adults. The relationship between sleep duration and chronic pain is complex and multifaceted. Research has demonstrated that insufficient sleep can exacerbate chronic pain sensitivity and contribute to the development of chronic pain conditions. For example, a systematic review of longitudinal studies revealed that a decline in sleep quality and quantity is associated with an increased risk of developing chronic pain conditions, which underscores the importance of sleep in the management of chronic pain-related health outcomes [36]. This review consolidates the evidence demonstrating that changes in sleep are prospectively associated with chronic pain-related outcomes. Moreover, chronic exposure to insufficient sleep has been found to alter the processes of chronic pain habituation and sensitization. In a study involving healthy adults, chronic sleep restriction was associated with increased chronic pain sensitivity over time. This effect was linked to changes in central chronic pain-modulatory processes, suggesting that chronic insufficient sleep may increase the vulnerability to chronic pain by altering the ability of the body to respond to it [37]. In contrast, other studies have revealed no significant association between sleep duration and chronic pain. One study discussing the bidirectional relationship among sleep duration, sleep disorders, and chronic pain suggested that while sleep disorders exacerbate chronic pain, the direct role of sleep duration in chronic pain remains unclear [38]. Another systematic review and meta-analysis examined the impact of changes in sleep duration on chronic pain-related health outcomes. That study emphasized that while poorer sleep quality and sleep duration are associated with an increased risk of developing chronic pain, the specific role of sleep duration in chronic pain remains unclear [39]. The inconsistent results regarding the relationship between sleep duration and chronic pain may be due to the differences in sample sizes, study populations, and study designs used across studies.

The relationship between sleep duration and chronic pain is complex and multifaceted, and it involves various physiological and psychological mechanisms. First, sleep disturbances, including insufficient sleep duration, have been shown to exacerbate pain sensitivity and interfere with pain modulation processes. Chronic exposure to insufficient sleep can alter the processes of pain habituation and sensitization, potentially increasing the vulnerability to chronic pain by affecting central pain-modulatory circuits. These findings suggest that the mechanisms that links sleep duration and chronic pain may involve changes in the central nervous system’s ability to regulate pain over time [40]. Second, total sleep deprivation has been found to increase pain sensitivity and impair conditioned pain modulation in healthy individuals. These findings indicate that sleep deprivation can disrupt descending pain pathways and facilitate spinal excitability, leading to heightened pain perception. These findings underscore the importance of getting adequate sleep to maintaining the body’s natural pain inhibition processes and suggest that sleep therapy might help normalize pain sensitivity in individuals who experience sleep deprivation [41]. Third, the bidirectional relationship between sleep and pain is highlighted by the fact that sleep deficiency can both cause and result from chronic pain. The neurobiological mechanisms involved in this relationship include the opioid, monoaminergic, and immune systems, among others. These systems play roles in the modulation of pain caused by sleep deficiency, suggesting that interventions that target these pathways could be beneficial in managing chronic pain as a comorbidity with sleep disturbances [11].

Our study revealed a positive association between the frailty index and chronic pain in different body regions, which is a relationship supported by previous research. One study linked higher frailty index scores to an increased risk of low back pain in middle-aged and older Chinese adults [42]. Another study suggested that chronic pain contributes to frailty, which then worsens the chronic pain [43]. Wade et al. also reported that chronic pain increases the risk of frailty in older adults [44]. However, Megale et al.‘s prospective cohort study did not find a significant link between frailty and chronic pain, possibly because of the small sample size and participant dropout rate of that study [45].

Frailty and chronic pain are interconnected conditions that often coexist in older adults and influence each other through complex mechanisms. One of the primary mechanisms through which frailty affects chronic pain is through the sensory, emotional, and cognitive aspects of pain perception. Frail individuals often experience heightened pain sensitivity and emotional distress, which can amplify their pain perception. This finding is supported by studies showing that frail older adults have worse scores for these aspects of pain than their nonfrail counterparts do, thereby suggesting that frailty can intensify the experience of chronic pain [46]. Genetic factors also play a significant role in the relationship between frailty and chronic pain. Research has demonstrated the presence of a shared genetic influence on both conditions, indicating that common genetic factors can predispose individuals to both frailty and chronic widespread pain. This shared genetic basis suggests that frailty and chronic pain might stem from similar underlying biological processes, which could include inflammation, hormonal changes, and neural degeneration [47]. The bidirectional relationship between frailty and chronic pain is further highlighted by studies that apply Mendelian randomization, which have shown that genetic predispositions to frailty are associated with an increased risk of chronic pain, and vice versa. This suggests that the presence of one condition can exacerbate the other, creating a vicious cycle that can be challenging to break [48, 49]. Moreover, chronic pain can lead to decreased physical activity, which is a critical component of frailty. Reduced physical activity due to pain can result in muscle weakness, decreased endurance, and further physical decline, all of which contribute to frailty. This decrease in physical activity not only affects physical health but also impacts social interactions and mental well-being, further compounding the effects of frailty [50]. The impact of frailty on chronic pain is also evident in the context of specific pain types, such as chronic lower back pain. Studies have shown that frailty status significantly influences the health-related quality of life in individuals with chronic lower back pain, with frail individuals experiencing more severe pain and lower quality of life than their robust counterparts. This underscores the importance of addressing frailty in managing chronic pain to improve overall health outcomes [51]. In conclusion, the relationship between frailty and chronic pain is complex and multifaceted, and it involves genetic, physiological, and psychosocial factors. Understanding these mechanisms is crucial for developing effective interventions for managing both conditions and improving the quality of life of older adults [52].

The potential mediating role of frailty in the association between sleep duration and chronic pain is supported by multiple studies. First, the interconnections among frailty, sleep quality, and chronic pain have been extensively investigated in the elderly population. Research has revealed a significant correlation between poor sleep quality and physical frailty, with frailty being closely linked to chronic pain [25]. Second, the bidirectional relationship between insufficient sleep and chronic pain is well documented. Insufficient sleep can heighten pain sensitivity, whereas chronic pain can lead to inadequate sleep, thereby creating a vicious cycle [8]. In addition, the impact of the duration of sleep on physical weakness should not be ignored. Research has shown that both overly short and overly long sleep durations might be associated with physical weakness, which is an important influencing factor for chronic pain [53].

The research presented above demonstrates a significant association among sleep duration, frailty index, and chronic pain across various body regions. The importance of investigating chronic pain in distinct body regions is underscored by the clinical and pathophysiological heterogeneity inherent to these conditions. This heterogeneity arises from variations in innervation, tissue types, and localized inflammatory or neurological processes, which leads to differing mechanisms for chronic pain. For example, back pain is frequently linked to structural degeneration, such as intervertebral disc protrusion, whereas pain in other regions may be associated with local inflammation and mechanical stress. Through an individual examination of pain sites, this study identifies region-specific risk factors, offering valuable insights for middle-aged and elderly populations in China and assisting in the prevention of chronic pain across different body regions.

This study has multiple strengths. First, it is a national study using a large and diverse sample, which makes its findings broadly applicable. Second, the sociodemographic factors and covariates were critically adjusted to mitigate any potential confounding effects. However, it is crucial to recognize this study’s limitations. First, the data on sleep duration and chronic pain in different body regions were obtained through self-report surveys, which may have led to reporting bias. In addition, owing to the limitations of the CHARLS database, the association with pain in a single, nonoverlapping site cannot be considered. Second, some subjects were excluded from the study on the basis of specific criteria, and this nonrandom selection may have introduced selection bias in the results. Third, our findings may have limited generalizability because our study included only middle-aged and older Chinese adults; this limitation may limit the relevance of our results, excluding other ethnicities or global populations. Fourth, we performed a mediation analysis using a cross-sectional study design, which posed challenges in establishing causal associations. Considering the limitations inherent to the present study, these results should be interpreted with caution, and further research is necessary to substantiate our findings.

## Conclusion

Our findings suggest that sleep duration is negatively associated with chronic pain, whereas the frailty index is positively associated with pain. Additionally, the frailty index mediates the relationship between sleep duration and chronic pain. Policies that promote sleep health and frailty prevention may indirectly reduce the chronic pain burden in ageing populations.

## Supplementary Information

Below is the link to the electronic supplementary material.

Supplementary Material 1

## Data Availability

No datasets were generated or analysed during the current study.
