# Supplementary material for: Mediation of the frailty index, the relationship between sleep duration and chronic pain in different body regions: insights from the CHARLS cross-sectional study
Source: J Orthop Surg Res. 2025 Aug 6;20:731. doi: 10.1186/s13018-025-06157-5 (PMC12326722; doi:10.1186/s13018-025-06157-5)
Supplement: Supplementary file 1 — Supplementary Material 1 [file 13018_2025_6157_MOESM1_ESM.doc]

**Frailty index mediates the relationship between sleep duration and chronic pain in different body regions**

**: insights from the CHARLS cross-sectional study**

**Supplementary materials**

**Supplemental Table 1.** Variables Used to Construct the Frailty Index.

**Supplemental Table 2.** Correlations among sleep duration, frailty index and pain

**Supplemental Table 3.** Threshold effect analysis of the relationship of sleep duration with headache

**Supplemental Table 4.** Threshold effect analysis of the relationship of sleep duration with shoulder pain

**Supplemental Table 5.** Threshold effect analysis of the relationship of sleep duration with wrist pain

**Supplemental Table 6.** Threshold effect analysis of the relationship of sleep duration with fingers pain

**Supplemental Table 7.** Threshold effect analysis of the relationship of sleep duration with chest pain

**Supplemental Table 8.** Threshold effect analysis of the relationship of sleep duration with stomachache

**Supplemental Table 9.** Threshold effect analysis of the relationship of sleep duration with back pain

**Supplemental Table 10.** Threshold effect analysis of the relationship of sleep duration with waist pain

**Supplemental Table 11.** Threshold effect analysis of the relationship of sleep duration with leg pain

**Supplemental Table 12.** Threshold effect analysis of the relationship of sleep duration with knees pain

**Supplemental Table 13.** Threshold effect analysis of the relationship of sleep duration with ankle pain

**Supplement Fig 1.Nonlinear associations of sleep duration and chronic pain in different body regions.** Solid and dashed lines represent the predicted value and 99% CIs, respectively. Orange bars represent the distribution of the entire cohort .Adjusted for age, gender, educational level, marital status, residence, smoking status, drinking status, BMI, and 14 chronic diseases,Only 99% of the data is displayed.

**Supplemental Table 1. Variables Used to Construct the Frailty Index.**

| **No** | **Description of the items** | **Cut-off value** |
| --- | --- | --- |
| 1 | Self-reported diagnosis of hypertension by a doctor | Yes = 1,  No = 0 |
| 2 | Self-reported diagnosis of diabetes by a doctor |
| 3 | Self-reported diagnosis of heart attack, coronary heart disease, angina, congestive heart failure, or other heart problems by a doctor |
| 4 | Self-reported diagnosis of stroke by a doctor |
| 5 | Self-reported diagnosis of cancer by a doctor |
| 6 | Self-reported diagnosis of arthritis by a doctor |
| 7 | Self-reported diagnosis of chronic lung diseases by a doctor |
| 8 | Self-reported diagnosis of asthma by a doctor |
| 9 | Self-reported diagnosis of emotional, nervous, or psychiatric problems by a doctor |
| 10 | Self-reported diagnosis of memory-related disease by a doctor |
| 11 | Self-reported vision problems |
| 12 | Self-reported hearing problems |
| 13 | Difficulty with dressing | Did not have any problems with the activity=0; some difficulty with the activity or could not do the activity=1. |
| 14 | Difficulty with bathing or showering |
| 15 | Difficulty with eating |
| 16 | Difficulty with getting in and out of bed |
| 17 | Difficulty with using the toilet |
| 18 | Difficulty with managing money |
| 19 | Difficulty with taking medications |
| 20 | Difficulty with shopping for groceries |
| 21 | Difficulty with preparing meals |
| 22 | Difficulty with doing housework |
| 23 | Difficulty with walking 100 yards |
| 24 | Difficulty with getting up from a chair after sitting for long periods |
| 25 | Difficulty with climbing several flights of stairs without resting |
| 26 | Difficulty with lifting or carrying weights over 10 pounds/ jins |
| 27 | Difficulty with picking up a coin from the table |
| 28 | Difficulty with stooping, kneeling, or crouching |
| 29 | Difficulty with reaching arms above shoulder level |
| 30 | Self reported health | Poor/ fair = 1, excellent/ very good/ or good = 0 |
| 31 | Depressive symptoms: CESD-10 questionnaire | CESD-10 >10 =1, ≤10 =0 |
| 32 | Cognition: (memory test score + orientation test score) **/** 14 | Continuous, ranging from 0 to 1 |
| Depression symptoms is evaluated using Center for Epidemiologic Studies Depression Scale (CESD). CESD-10 is used, and the total score ranges from 0 to 30.  The memory score is calculated as the average of words that are not remembered in the immediate and delayed word recall tasks. The memory score ranges from 0 to 10. The orientation test consists of four four questions about the day of the week, the month, the date of the month, and the year. Each incorrect response earns one point, resulting in a scoring range from 0 to 4. Thus, the sum of memory and orientation test scores was divided by 14 to yield a continuous cognitive variable, ranging from 0 to 1. | | |

**Supplemental Table 2. Correlations among sleep duration, frailty index and chronic pain in different body regions.**

| Variables | Sleep duration | Frailty index | Headache | Shoulder pain | Arm pain | Wrist pain | Fingers pain | Chest pain | Stomachache | Back pain | Waist pain | Buttocks pain | Leg pain | Knees pain | Ankle pain | Toes pain | Neck pain |
| --- | --- | --- | --- | --- | --- | --- | --- | --- | --- | --- | --- | --- | --- | --- | --- | --- | --- |
| Sleep duration | 1.00 |  |  |  |  |  |  |  |  |  |  |  |  |  |  |  |  |
| Frailty index | -0.23^***^ | 1.00 |  |  |  |  |  |  |  |  |  |  |  |  |  |  |  |
| Headache | -0.17^***^ | 0.39^***^ | 1.00 |  |  |  |  |  |  |  |  |  |  |  |  |  |  |
| Shoulder pain | -0.16^***^ | 0.39^***^ | 0.58^***^ | 1.00 |  |  |  |  |  |  |  |  |  |  |  |  |  |
| Arm pain | -0.16^***^ | 0.38^***^ | 0.53^***^ | 0.64^***^ | 1.00 |  |  |  |  |  |  |  |  |  |  |  |  |
| Wrist pain | -0.13^***^ | 0.34^***^ | 0.45^***^ | 0.56^***^ | 0.60^***^ | 1.00 |  |  |  |  |  |  |  |  |  |  |  |
| Fingers pain | -0.15^***^ | 0.36^***^ | 0.45^***^ | 0.54^***^ | 0.56^***^ | 0.62^***^ | 1.00 |  |  |  |  |  |  |  |  |  |  |
| Chest pain | -0.13^***^ | 0.32^***^ | 0.45^***^ | 0.44^***^ | 0.45^***^ | 0.43^***^ | 0.43^***^ | 1.00 |  |  |  |  |  |  |  |  |  |
| Stomachache | -0.14^***^ | 0.3^***^ | 0.50^***^ | 0.48^***^ | 0.44^***^ | 0.40^***^ | 0.39^***^ | 0.43^***^ | 1.00 |  |  |  |  |  |  |  |  |
| Back pain | -0.14^***^ | 0.36^***^ | 0.51^***^ | 0.61^***^ | 0.54^***^ | 0.50^***^ | 0.50^***^ | 0.46^***^ | 0.47^***^ | 1.00 |  |  |  |  |  |  |  |
| Waist pain | -0.17^***^ | 0.42^***^ | 0.55^***^ | 0.60^***^ | 0.55^***^ | 0.48^***^ | 0.48^***^ | 0.40^***^ | 0.49^***^ | 0.58^***^ | 1.00 |  |  |  |  |  |  |
| Buttocks pain | -0.12^***^ | 0.30^***^ | 0.38^***^ | 0.45^***^ | 0.45^***^ | 0.43^***^ | 0.42^***^ | 0.35^***^ | 0.35^***^ | 0.45^***^ | 0.44^***^ | 1.00 |  |  |  |  |  |
| Leg pain | -0.16^***^ | 0.43^***^ | 0.48^***^ | 0.56^***^ | 0.55^***^ | 0.49^***^ | 0.49^***^ | 0.39^***^ | 0.44^***^ | 0.53^***^ | 0.61^***^ | 0.45^***^ | 1.00 |  |  |  |  |
| Knees pain | -0.16^***^ | 0.43^***^ | 0.55^***^ | 0.58^***^ | 0.56^***^ | 0.52^***^ | 0.51^***^ | 0.43^***^ | 0.46^***^ | 0.52^***^ | 0.60^***^ | 0.43^***^ | 0.53^***^ | 1.00 |  |  |  |
| Ankle pain | -0.14^***^ | 0.37^***^ | 0.43^***^ | 0.48^***^ | 0.50^***^ | 0.53^***^ | 0.51^***^ | 0.39^***^ | 0.37^***^ | 0.45^***^ | 0.46^***^ | 0.43^***^ | 0.52^***^ | 0.53^***^ | 1.00 |  |  |
| Toes pain | -0.12^***^ | 0.33^***^ | 0.36^***^ | 0.41^***^ | 0.42^***^ | 0.44^***^ | 0.50^***^ | 0.36^***^ | 0.31^***^ | 0.40^***^ | 0.37^***^ | 0.39^***^ | 0.43^***^ | 0.43^***^ | 0.54^***^ | 1.00 |  |
| Neck pain | -0.13^***^ | 0.34^***^ | 0.52^***^ | 0.61^***^ | 0.54^***^ | 0.50^***^ | 0.49^***^ | 0.42^***^ | 0.44^***^ | 0.54^***^ | 0.52^***^ | 0.41^***^ | 0.49^***^ | 0.51^***^ | 0.45^***^ | 0.39^***^ | 1.00 |

**Supplemental Table 3. Threshold effect analysis of the relationship of sleep duration with headache.**

| sleep duration | Adjusted Model | |
| --- | --- | --- |
|  | OR (95% CI) | P value |
| < 6.57 | 0.72(0.679~6.69) | <0.001 |
| ≧6.57 | 0.98(0.89~1.08) | 0.75 |
| Likelihood Ratio test |  | <0.001 |
| Adjusted for age, gender, educational level, marital status, residence, smoking status, drinking status, BMI, and 14 chronic diseases, Only 99% of the data is displayed. Abbreviations: OR, odds ratio; 95% CI, 95% confidence interval. | | |

**Supplemental Table 4. Threshold effect analysis of the relationship of sleep duration with shoulder pain.**

| sleep duration | Adjusted Model | |
| --- | --- | --- |
|  | OR (95% CI) | P value |
| < 7.46 | 0.79(0.75~0.82) | <0.001 |
| ≧7.46 | 0.95(0.82~1.08) | 0.41 |
| Likelihood Ratio test |  | <0.001 |
| Adjusted for age, gender, educational level, marital status, residence, smoking status, drinking status, BMI, and 14 chronic diseases, Only 99% of the data is displayed. Abbreviations: OR, odds ratio; 95% CI, 95% confidence interval. | | |

**Supplemental Table 5. Threshold effect analysis of the relationship of sleep duration with wrist pain.**

| sleep duration | Adjusted Model | |
| --- | --- | --- |
|  | OR (95% CI) | P value |
| < 8.21 | 0.83(0.79~0.83) | <0.001 |
| ≧8.21 | 1.17(0.84~1.64) | 0.36 |
| Likelihood Ratio test |  | <0.001 |
| Adjusted for age, gender, educational level, marital status, residence, smoking status, drinking status, BMI, and 14 chronic diseases, Only 99% of the data is displayed. Abbreviations: OR, odds ratio; 95% CI, 95% confidence interval. | | |

**Supplemental Table 6. Threshold effect analysis of the relationship of sleep duration with fingers pain.**

| sleep duration | Adjusted Model | |
| --- | --- | --- |
|  | OR (95% CI) | P value |
| < 8.11 | 0.82(0.79~0.86) | <0.001 |
| ≧8.11 | 1.14(0.81~1.61) | 0.45 |
| Likelihood Ratio test |  | <0.001 |
| Adjusted for age, gender, educational level, marital status, residence, smoking status, drinking status, BMI, and 14 chronic diseases, Only 99% of the data is displayed. Abbreviations: OR, odds ratio; 95% CI, 95% confidence interval. | | |

**Supplemental Table 7. Threshold effect analysis of the relationship of sleep duration with chest pain.**

| sleep duration | Adjusted Model | |
| --- | --- | --- |
|  | OR (95% CI) | P value |
| < 8.41 | 0.79(0.78~0.84) | <0.001 |
| ≧8.41 | 1.03(0.69~1.53) | 0.87 |
| Likelihood Ratio test |  | <0.001 |
| Adjusted for age, gender, educational level, marital status, residence, smoking status, drinking status, BMI, and 14 chronic diseases, Only 99% of the data is displayed. Abbreviations: OR, odds ratio; 95% CI, 95% confidence interval. | | |

**Supplemental Table 8. Threshold effect analysis of the relationship of sleep duration with stomachache.**

| sleep duration | Adjusted Model | |
| --- | --- | --- |
|  | OR (95% CI) | P value |
| < 6.68 | 0.78(0.73~0.82) | <0.001 |
| ≧6.68 | 0.94(0.83~1.05) | 0.27 |
| Likelihood Ratio test |  | 0.006 |
| Adjusted for age, gender, educational level, marital status, residence, smoking status, drinking status, BMI, and 14 chronic diseases, Only 99% of the data is displayed. Abbreviations: OR, odds ratio; 95% CI, 95% confidence interval. | | |

**Supplemental Table 9. Threshold effect analysis of the relationship of sleep duration with back pain.**

| sleep duration | Adjusted Model | |
| --- | --- | --- |
|  | OR (95% CI) | P value |
| < 7.28 | 0.79(0.66~0.84) | <0.001 |
| ≧7.28 | 0.83(0.73~1.28) | 0.56 |
| Likelihood Ratio test |  | 0.001 |
| Adjusted for age, gender, educational level, marital status, residence, smoking status, drinking status, BMI, and 14 chronic diseases, Only 99% of the data is displayed. Abbreviations: OR, odds ratio; 95% CI, 95% confidence interval. | | |

**Supplemental Table 10 Threshold effect analysis of the relationship of sleep duration with waist pain.**

| sleep duration | Adjusted Model | |
| --- | --- | --- |
|  | OR (95% CI) | P value |
| < 8.22 | 0.83(0.81~0.86) | <0.001 |
| ≧8.22 | 0.81(0.63~1.04) | 0.10 |
| Likelihood Ratio test |  | 0.01 |
| Adjusted for age, gender, educational level, marital status, residence, smoking status, drinking status, BMI, and 14 chronic diseases, Only 99% of the data is displayed. Abbreviations: OR, odds ratio; 95% CI, 95% confidence interval. | | |

**Supplemental Table 11. Threshold effect analysis of the relationship of sleep duration with Leg pain.**

| sleep duration | Adjusted Model | |
| --- | --- | --- |
|  | OR (95% CI) | P value |
| < 8.23 | 0.83(0.8~0.86) | <0.001 |
| ≧8.23 | 0.81(0.63~1.04) | 0.38 |
| Likelihood Ratio test |  | 0.01 |
| Adjusted for age, gender, educational level, marital status, residence, smoking status, drinking status, BMI, and 14 chronic diseases, Only 99% of the data is displayed. Abbreviations: OR, odds ratio; 95% CI, 95% confidence interval. | | |

**Supplemental Table 12. Threshold effect analysis of the relationship of sleep duration with knees pain.**

| sleep duration | Adjusted Model | |
| --- | --- | --- |
|  | OR (95% CI) | P value |
| < 7.75 | 0.79(0.76~0.84) | <0.001 |
| ≧7.75 | 1.01(0.89~1.15) | 0.86 |
| Likelihood Ratio test |  | 0.001 |
| Adjusted for age, gender, educational level, marital status, residence, smoking status, drinking status, BMI, and 14 chronic diseases, Only 99% of the data is displayed. Abbreviations: OR, odds ratio; 95% CI, 95% confidence interval. | | |

**Supplemental Table 13. Threshold effect analysis of the relationship of sleep duration with ankle pain.**

| sleep duration | Adjusted Model | |
| --- | --- | --- |
|  | OR (95% CI) | P value |
| < 6.56 | 0.75(0.69~0.80) | <0.001 |
| ≧6.56 | 0.91(0.79~1.03) | 0.14 |
| Likelihood Ratio test |  | 0.009 |
| Adjusted for age, gender, educational level, marital status, residence, smoking status, drinking status, BMI, and 14 chronic diseases, Only 99% of the data is displayed. Abbreviations: OR, odds ratio; 95% CI, 95% confidence interval. | | |

**
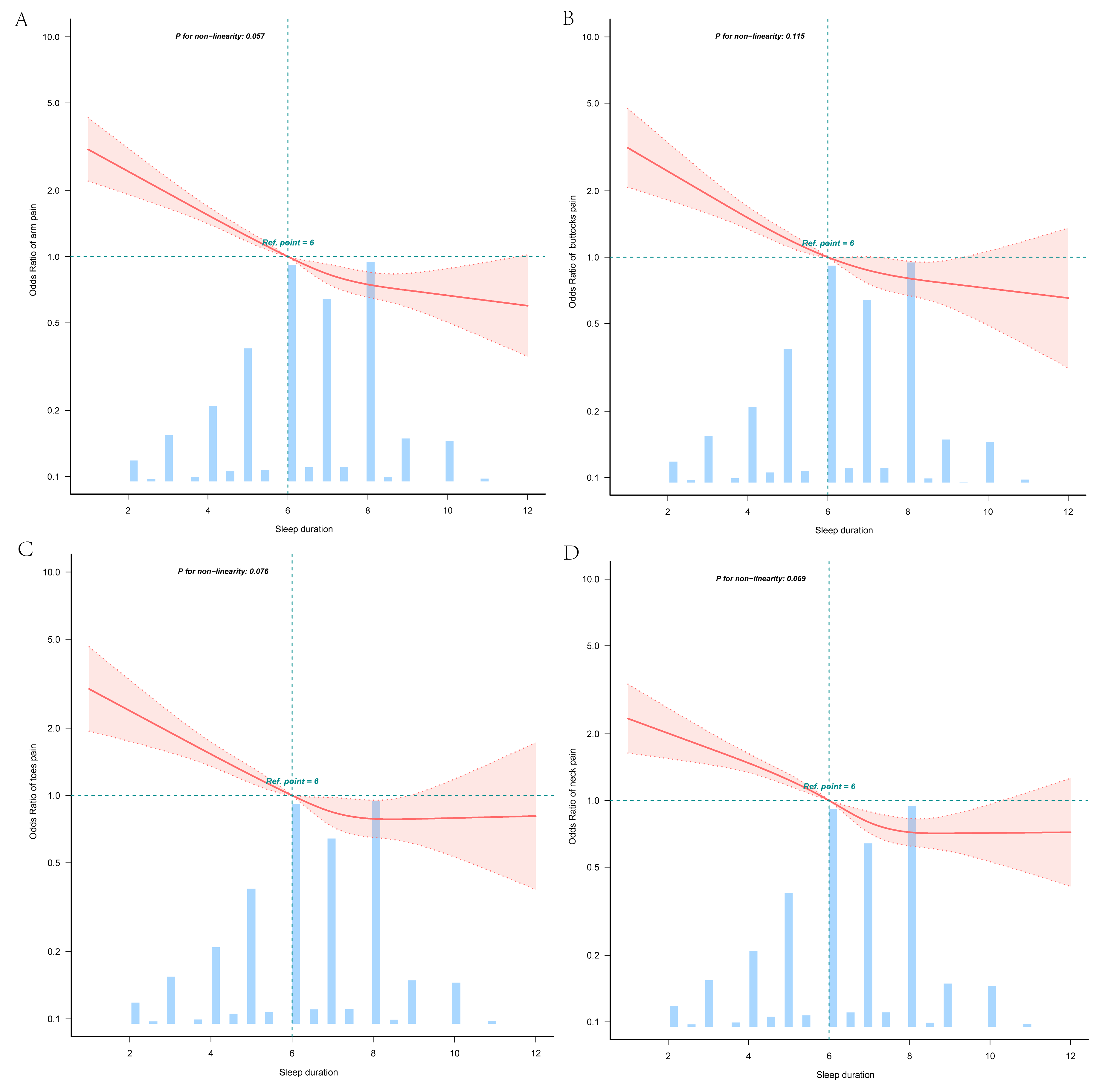
**

**Supplement Fig 1.Nonlinear associations of sleep duration and chronic pain in different body regions.** Solid and dashed lines represent the predicted value and 99% CIs, respectively. Orange bars represent the distribution of the entire cohort .Adjusted for age, gender, educational level, marital status, residence, smoking status, drinking status, BMI, and 14 chronic diseases,Only 99% of the data is displayed.
